# Video Relay Service Interpreters’ Experiences with Caller Behavior: An Occupational Health Risk Call to Action

**DOI:** 10.3390/healthcare13172116

**Published:** 2025-08-26

**Authors:** Robyn K. Dean, Catherine Cerulli, Daniel J. Devor, Robert Q Pollard, Jr., Sarah E. Biello, Daniel Maffia, Hugh F. Crean

**Affiliations:** 1 Rochester Institute of Technology, National Technical Institute for the Deaf, 52 Lomb Memorial Dr., Rochester, NY 14623, USA; danny.maffia@rit.edu; 2 Department of Psychiatry, University of Rochester School of Medicine and Dentistry, 300 Crittenden Blvd., Rochester, NY 14642, USArobert_pollard@urmc.rochester.edu (R.Q.P.J.); hugh_crean@urmc.rochester.edu (H.F.C.); 3 Independent Researcher, Greenville, SC 29601, USA; 4 Department of Translation and Interpreting, Gallaudet University, 800 Florida Avenue, NE, Washington, DC 20002, USA; sarah.biello@gallaudet.edu

**Keywords:** signed language interpreters, video relay service, VRS companies, occupational health, stress, FCC policies

## Abstract

**Background/Objectives**: Research raising concerns about the occupational health of signed language interpreters has proliferated in the past two decades. Recent studies examining interpreters’ various work settings find that Video Relay Service (VRS) work is linked to greater health risks than other interpreting settings. This study aimed to shed light on why VRS work appears to be particularly hazardous. **Methods**: This mixed-methods study reports data from an online survey of 345 American VRS interpreters. Participants were queried about a range of potentially stressful experiences with callers. Quantitative data regarding the types, frequency, patterns, and consequences of stressful calls were further informed by qualitative data reported by participants in free-response survey fields. **Results**: Incidents of VRS interpreters mediating calls regarding sexual activity, drug deals, and prostitution were reported with notable frequency, as was interpreters’ witnessing abuse of vulnerable individuals. Interpreters also were often the object of callers’ derisive sexual, physical, and racial comments. Yet the incidence of participants reporting these experiences to management or outside authorities was quite limited despite the potential legal jeopardy involved. When reports were made, most participants stated their companies took little or no action. We also examined how factors such as the tenure of VRS, hours worked per week, and work shift times were associated with such caller experiences. **Conclusions**: This study builds upon prior VRS health risk research by examining external factors, including caller behavior and employer policies, that may contribute to interpreter stress and burnout. Suggestions for remediation and workforce development, involving VRS companies, the Federal Communications Commission (FCC) and state legislation are offered.

## 1. Introduction

There is a video that can be readily found on the Internet of a person whose face is completely covered by a ghost-like mask with distorted eyes and a gaping mouth; it is the mask readily recognized from the American movie, *Scream*. The video contains no sound but features a real-time interaction of a video call to a signed language interpreter in professional attire, wearing a headset, working for a Video Relay Service (VRS). The masked signer uses American Sign Language (ASL) to direct the VRS interpreter to call 911 to order a pizza—clearly a prank call.

This 16 s clip is part of a nearly three-minute video that includes similar calls placed to four other VRS interpreters. In each segment, the signer is masked. The nature of the calls escalates from the absurd (e.g., pizza delivery) to the derisive (e.g., insulting 911 personnel) to the profane (e.g., asking for police to be sent to “s*ck his d**k”).

The video captures the faces and upper bodies of all four interpreters—presumably women—who appear resigned to interpret these obscene and likely illegal calls. Despite the clearly offensive content, the VRS interpreters connect and process the calls as they are required to do under United States (US) Federal Communications Commission (FCC) regulations. That is, all calls must be relayed regardless of the nature of the call. The VRS interpreters were recorded without their consent, and the footage was widely shared online.

Prank calls to 911 are illegal and can be prosecuted as misdemeanors or felonies, depending on state laws and the severity of the call. However, tracing the caller in this case is complicated. The masked signer is calling 911 indirectly, through a third party’s phone system, a VRS company. At the time this interaction was recorded, determining the location of the originating videophone caller would have either been impossible or resulted in significant delays. This creates a system ripe for abuse. Callers like the masked signer know that interpreters are legally obligated to process their calls and that their own identities can remain hidden. As the masked signer tells one of the interpreters, “You don’t know where I live.”

This viral video spotlights several important elements of the current study. The first is the use of technology in the delivery of interpreting services. This federally funded service provided to deaf and hard-of-hearing Americans relies on videophone technology and the vast, existing telecommunications network in the US. Second, it highlights some of the problematic call content VRS interpreters must navigate—from simple pranks to serious emergencies, to calls involving illegal activity, and more. Finally, it raises urgent questions about interpreter working conditions and the responsibility of VRS companies to protect employees from legal, emotional, and psychological harm. This study was conducted to investigate the degree to which VRS interpreters are exposed to aggressive, even abusive, calls and whether interpreters are adequately prepared, supported, and, when necessary, shielded from the potentially harmful or illegal nature of certain VRS calls. We pursued these questions through a mixed-method research procedure surveying VRS interpreters throughout the US.

### 1.1. Interpreting and Technology

Technology has played a crucial role in the delivery of interpreting services, dating back almost 100 years. This technology was brought to the world stage at the Nuremberg Trials in the mid-1940s [1,2]. A century later, interpreters in conference settings use the same basic technology—audio input and output via headphones and microphones, just as with their earliest predecessors. Furthermore, interpreting over the telephone dates back to 1957, which was employed in conference settings. Over-the-phone interpreting (OPI) marks the first example of remote interpreting. Remote interpreters are separated from their interlocutors, whether by a few hundred feet or a few thousand miles, but connected through videoconferencing technology or the like [3].

In community interpreting (interpreting in medical, educational, legal, and other settings), OPI began in the US in the early 1980s and even earlier in other parts of the world [4]. However, around the turn of this century, there was a steep rise in the technology-based delivery of interpreting services, including the use of videoconferencing technologies [3,5,6,7]. This was further amplified during the COVID-19 global pandemic when interpreters, like many workers around the world, shifted to remote work [7,8].

Whether interpreters are working between two spoken languages or when one of those languages is a signed language, the move to remote interpreting has exacerbated the already complex and fatiguing task of interpreting between two languages [2,3,9,10]. While the use of technology has long been a significant factor in the work of interpreters, more recent technological developments and the real-time pressures and near-constant availability that technology affords present new concerns for worker health and sustainability [7,8,9,10,11,12].

There is ample and long-standing research documenting the stress experienced by both signed and spoken language interpreters [13,14,15,16,17,18,19,20,21]. The earliest research on interpreter stress investigated in-person (on-site) interpreting in both conference and community settings [13,19]. For signed language interpreters, much of the early research focused on physical pain and injury due to the repetitive motion of the upper body and extremities used in signed languages [22]. With the rise in remote interpreting, greater attention is now being paid to the broader well-being of interpreters working remotely [3,5,10].

In one study comparing on-site and remote interpreting, 36 spoken language interpreters reported that remote interpreting created a whole new set of demands in their work [2]. Study participants reported that remote interpreting resulted in problematic physical demands such as screen glare and poor visibility of interlocutors, resulting in insufficient feedback cues. They also reported problematic psychological factors such as a sense of isolation, alienation, and an overall loss of control [6,11].

Roman et al. used a quantitative approach to study the physical and psychological impact on signed language interpreters working remotely across setting domains. Employing several standard measures of physical and psychological health, Roman and colleagues found a positive association between participants’ physical and psychological health. A pain intensity instrument showed that participants reported elevated baseline pain levels mostly affecting the upper body and extremities. The overall measures of mental health were within normal limits, but the researchers noted that over one-third of respondents scored above the norm on at least one mental health subscale, including scales of depression, anxiety, and stress [10].

### 1.2. Working Conditions of Interpreters

The introduction of technology in interpreting services ushered in another marked shift in how interpreters work today—simultaneous interpreting, the task of listening and speaking at the same time [23]. The trials at Nuremberg put simultaneous interpreting and the technology that allows for its provision on the world stage [1,24]. Unlike translators who work with written text, Xing noted that simultaneous interpreters are processing high-density information that cannot be reviewed nor is there much of a chance to revise the target language once it is produced for the end-users [25]. While the task of translating between two languages, in general, is cognitively complex, simultaneous interpreting requires additional effort and pressure to process and produce an accurate interpretation in real-time [23,24,25,26,27].

The simultaneous task of delivering the target language while the source language is being received leads to cognitive saturation [9]. Given the cognitive load and fatigue of simultaneous interpreting, studies have shown that interpreting performance degrades after twenty to thirty minutes of continuous work [9,13,23,28]. As a result, it is generally accepted that interpreters should work in teams of two or three and should switch off every twenty minutes for a needed cognitive break [2,9,18]. For signed language interpreters, this break may also be needed for recovery from the physical toll of signing, and specifically signing to a camera that can only capture a limited field of view (i.e., restricting larger signed movements to stay within the frame). Given these additional demands of remote interpreting, it has been recommended that interpreters’ time on tasks be reduced by half the normal standard. In other words, remote, simultaneous interpreters should only work continuously for ten to fifteen minutes at a time [3].

Interpreters working in remote settings likely do not work for ten- to fifteen-minute intervals. Companies that employ interpreters in call centers (or in-home offices) likely could not sustain a viable business with interpreters working one call in teams of two or three. In VRS, interpreters are typically expected to work a full 50 min in order to earn a ten-minute break [28]. Inadequate break times and high call volumes were blamed for VRS interpreters’ stress and burnout in a study of 424 VRS interpreters [28]. The online survey asked VRS interpreters to rate 20 stress factors, 15 of which were rated as significantly stressful. The top-rated stress factors included angry callers, concerns about the brief length of time between calls, concerns about physical strain, processing emergency 911 calls, and interpreting calls with limited contextual information.

Alley interviewed 20 VRS interpreters who echoed many of the concerns already mentioned, including restricting autonomy or decision latitude. One finding in this study revealed why interpreters might comply with the perceived rigid policies of their employers. VRS interpreters are technologically monitored, and metrics are collected (e.g., non-billable minutes such as time in between calls). VRS interpreters reported that employers use these efficiency benchmarks to reward employees (e.g., offering preferred schedules) and to penalize them (e.g., reducing shift hours). The participants acknowledged that they are often compliant with policies not out of agreement with the rules or company loyalty but as a means to an end—obtaining their preferred shift [5].

Olopade found some of the same concerns when surveying the experiences of 34 Black/African American VRS interpreters. While some concerns were echoed in other qualitative VRS studies (e.g., heavy workload, restrictive company policies, effects on health and mental health), Black/African American interpreters reported being the subject of disturbing, racially charged comments and behaviors from callers (e.g., “I don’t want a Black interpreter”). Some participants even reported similar microaggressions from their call center colleagues (e.g., asking for tips on how to use “Black slang”). Participants further reported that support from management was lacking, conveying a greater concern for profit-based metrics than employee well-being [29]. Lack of management support and microaggressions against VRS interpreters who are (or were perceived to be) lesbian, gay, bisexual, transgender, and queer (LGBTQ+) were also key findings of Donovan’s study [30].

As much of the qualitative and quantitative data on VRS interpreters are self-reported in nature, Pollard and colleagues instead sought biological evidence of reported stress and burnout. Pollard et al. measured the daily rhythm of salivary cortisol levels of signed language interpreters working across three primary work settings: general community settings, VRS, and primary and secondary educational settings. VRS interpreters were the only participant group of this study working remotely [31]. The biological data corroborated prior self-report data that showed that VRS interpreters and educational interpreters experience the highest occupational health risks, chronic stress, and burnout [31,32]. Furthermore, the self-report measures included in this study showed that the possible reasons for these poor occupational health outcomes were different for each participant group. For educational interpreters, participants reported greater concern over perceived competency. For VRS interpreters, participants reported greater concerns over autonomy [5,33].

### 1.3. Remote Interpreters’ Agency, Autonomy, and Call Content

The interpreter’s sense of agency or their presence in a virtual encounter has been identified in research as a key area of concern [2,34,35]. Interpreters working remotely report a sense of professional isolation and loneliness [10,36]. The effects of long-term disconnection may be contributing to higher-than-normal incidents of depression and anxiety found in interpreter participants [10,11,32,36]. Some remote interpreters have reported a sense of depersonalization, detachment, and cynicism [37], which are all well-established symptoms of burnout. In addition to the prevalence of interpreter stress research, burnout, vicarious trauma, and early departure from the field have been frequently reported [7,11,17,21,28,38]. Cortisol dysregulation and specifically a blunted cortisol awakening response are the biological indicators of burnout, which was identified in interpreters working remotely [2,29,37] and specifically in VRS [31].

The experience of decreased feelings of agency have further resulted in a sense of limited autonomy or decision latitude of remote interpreters. This decrease in autonomy is particularly problematic given the increased demands of remote work [3], and the even greater demands in VRS interpreting [32,33,39]. Certainly, this limitation in decision latitude or the loss of control could be a self-imposed limitation. However, others have argued that it is very much a reality, given the mandates and policies imposed by regulators and employers [5,31,40].

Spoken and signed language interpreters can find themselves in all circumstances of social and private life—from the grounds of school campuses to the fields of armed conflicts. While remote interpreting might offer some protection from the physical harm of more extreme working conditions, it is not a shield from psychological harm. Moreover, the potential for psychological harm increases as a result of the sheer volume and the rapid, continuous nature of processing calls at remote work sites. Indeed, the push to keep processing calls one right after the other can lead interpreters to feel like they are factory workers on an assembly line [37,40] without much support or recognition of their work and its complexity [8,28]. Examples of the types of stressful content reported by remote interpreters include death notifications [28]; sexual assault testimony [7]; oncology appointments for parents of children on a pediatric unit [9]; uncivil, fraudulent, and abusive behaviors between interlocutors [5]; and callers’ oppressive and discriminatory behaviors [12].

Some employers have put measures in place for remote interpreters to address stress they may experience as a result of difficult calls, such as peer or professional counseling [41]. Several studies found that access to these services was a barrier [5,29]. One study found that 93% of the 889 VRS interpreters surveyed did not seek the services of professional psychologists made available by their employers [12].

As documented above, the types of call content in remote interpreting can be of a highly sensitive and intimate nature. However, for signed language interpreters working in VRS settings, the types of call content have greater variety, and the levels of sensitivity are consequently amplified. When a medical provider does not share a language with their patient, remote interpreting is a frequent solution. While in-person or over-the-phone conversations between medical providers and patients can be highly sensitive and potentially stressful for a remote interpreter, that conversation will be structured around the medical topic at hand. Deaf and hard-of-hearing individuals who use sign language not only do not share a language with their medical provider but they also may not share a language with their family members and many relevant others.

Accordingly, what one says to their family or significant other in a phone call has fewer boundaries than what is said in a phone call in a specific healthcare context. As such, in addition to calls that concern pleasant or unpleasant family matters, VRS interpreters also must relay calls between an array of unrelated others, including calls between a drug dealer and their client and a sex worker and theirs [5].

Relaying calls from a videophone to an audio-only phone increases concerns for signed language interpreters because interpreters have visual access to the signer and their environment, which is not seen by the other hearing party. In addition to the shared logistical complaints of poor video quality, lighting, background noise, etc., VRS interpreters also report disruptive and disturbing visuals, such as witnessing child abuse and incidents of domestic violence [31]. They also report being confronted with partially clad or completely naked individuals, including a caller intentionally exposing their genitals to the camera [28]. Furthermore, emergency calls to 911 or a doctor’s office might require the interpreter to give detailed visual explanations of what they are seeing in order for aid to be given [5].

Another unique feature of interpreting in VRS is that all parties are in different locations. Before a call is connected to the desired end user, there is one-on-one engagement between the initiating caller (hearing or deaf) and the VRS interpreter. It is this feature of VRS interpreting that leads to some of the disturbing and abusive incidents reported in several studies, including racist comments, harsh critical comments, and direct sexual or lewd comments or solicitations, including callers masturbating on screen [5,28,29,30].

VRS interpreters were once able to use electronic “privacy screens,” a videophone feature that can be likened to turning off one’s camera during a videoconference call. Interpreters’ privacy screens could be used during a call (such as during significant wait times) or before, after, or in-between calls when there was no communication need for visual contact with the interpreter. While there are a few exceptions, VRS interpreters are now no longer permitted to use privacy screens [42]. However, callers using sign language can still use their privacy screen. This creates a powerful differential—a deaf caller now can see the interpreter at all times, but the interpreter cannot always see the deaf caller. A VRS interpreter must wait a full five minutes, while they are on camera but unable to see or solicit the attention of the deaf caller before they are permitted to disconnect the call [42].

VRS interpreters can only disconnect a call immediately if they realize the situation involves sexual abuse or exploitation of a child. In such cases, VRS interpreters also must make a report to the National Center for Missing and Exploited Children (NCMEC), per federal law (18 U.S.C. 2258A). However, they are prohibited from reporting any other type of child abuse they might witness to authorities. They are also instructed to not report elder abuse, incidents of domestic violence, threats of suicide (verbal or behavioral), or other conversations of a sexual nature that do not involve the exploitation of children. VRS interpreters are encouraged to reach out to their supervisor or to EAP counseling services to debrief after upsetting calls, but limits are placed on how much call content can be revealed. Debriefing is designed to be a support mechanism only, not official reporting with expected follow-up actions, although, according to Alley, more formal complaints can be made to VRS employers [5].

Much of the decision authority VRS interpreters have when dealing with calls that are inappropriate, harassing, or abusive is necessarily after the fact, after the call is completed and after any harm toward the interpreter has been incurred [29]. There appear to be few options during a call other than calling for a team interpreter or transferring the call for another colleague to deal with. Exercising either option will be measured as part of the interpreter’s efficiency and productivity metrics [29].

To this point in our review, the realities of and concerns for both spoken and signed language interpreters working remotely have been interwoven. The focus for the remainder of the current study is on VRS interpreting, which, for now, is an issue facing only signed language interpreters in the US and in some other countries. Granted, there are large-scale employers of remote interpreters of spoken and signed languages numbering in the thousands (e.g., Language Line), but the unique provisional and regulatory relationship with the US’s Federal Communication Commission (FCC) sets VRS interpreting apart from remote spoken language interpreting. As such, the remainder of this article focuses on VRS interpreting and its stakeholders—the FCC, VRS companies, deaf and hearing callers who access the service, and the thousands of employed VRS interpreters.

This is not to suggest that the concerns raised here are uniquely an American problem. The two largest VRS companies (who have 80% of the market share) that started operations in the US are now operating in other parts of the world. The Private Equity Stakeholder Project (PESP) is a nonprofit watchdog organization that monitors the private equity and broader private funds industry. According to the PESP report, Sorenson (owned by Ariel Alternatives) is the “world’s leading provider of communication tools for Deaf and hard-of-hearing people, with over 10,000 employees worldwide” [43] (p. 13). Additionally, Kinderhook-owned “ZP Better Together” has operations in Brazil, Canada, Germany, India, Japan, the Philippines, and the United Kingdom [44].

### 1.4. VRS Companies’ Profits and Policies

When hearing individuals need to make a call, they rely on audio-based technology—the microphone and the speaker of a mobile phone or a traditional land-line phone. When a deaf or hard-of-hearing person who primarily uses ASL or another signed language needs to make or receive a call, they need a videophone (VP). Videophone technologies can be likened to more familiar videoconferencing platforms such as Zoom and Microsoft Teams. A videophone can directly call another VP, but a VP cannot connect to a landline or a mobile phone. These devices do not share the same technology and most often, the persons on either end of the call do not share the same language. They need an interpreter who has access to both technologies and both languages who can relay the call.

In response to the mandates of Title IV of the Americans with Disabilities Act (ADA), the FCC must provide equal access to the US telecom network for persons with hearing or speech disabilities. The FCC’s broader Telecommunications Relay Services (TRS) is the entity that provides this access 24 h a day, 7 days a week, 365 days a year. The TRS fund compensates TRS providers (including all VRS providers, via the FCC) by obtaining these funds through surcharges on all telephone, cable, and Internet bills [45].

To comply with this legal mandate, the FCC contracts with VRS companies, who employ and manage a staff of interpreters, provide free access to videophones to deaf and hard-of-hearing sign language users across the country, and support the ongoing technological needs for this nationwide connectivity [46]. The FCC reimburses contracted VRS providers for each minute a videophone is connected to a non-videophone number. Reimbursement rates are tiered and based on the average number of minutes each company processes per month. At the time of this publication, reimbursement rates range between approximately USD 4 to USD 8 a minute [43].

There are two private equity-owned companies (a duopoly) that dominate the VRS market and account for over 80% of the national market share. One of those companies, Sorenson Communications, is now the largest employer of signed language interpreters in the US and, presumably, the world. The VRS industry is a multi-million-dollar business, with reports of per-minute reimbursements of USD 600 million annually [47].

Peterson sounded early alarm bells for what in essence is the corporate takeover of a once community-based, public service [40]. The myriad problems of this public-turned-private enterprise has, a full decade later, garnered outside attention. The PESP issued a report of its investigation into the VRS duopoly and identified some of the same interpreter concerns noted above: physical and mental strain, job dissatisfaction, insufficient breaks and support from management, and the sometimes disturbing, even abusive, nature of the calls and callers. The report also found additional concerns—evidence of union busting efforts, infrequent and low increases in hourly pay, use of forced arbitration in employee disputes, and gaps in FCC oversight [43].

The FCC gaps identified by the PESP report included delayed review of VRS companies’ operational certificates, lack of transparency regarding company finances (particularly debt loads, a unique feature of private equity), providers exempting customer complaints and performance information from disclosure, hindering public access to information about service quality, and concerns about the efficiency of a system where significant funds are spent on marketing to “poach” customers from other providers [43].

These concerns and others already noted herein were brought to the attention of the FCC in March 2025 with the help of Texas Congressman Greg Casar. Casar, who became concerned over frequent labor disputes between VRS interpreters and VRS companies, said during a press conference about the FCC telecommunications system, “there is plenty of money to do it. The question is, is it going to go to the interpreters and their clients… or is it going to go to the profits of a few extra-wealthy people?” [47]. It is the spirit of Casar’s question that compelled the current study. Part of our aim was to call attention to the mediating role that private-equity companies play in VRSs, positioned between a public, governmental entity (the FCC) and its public servants, the signed language interpreters.

Several of the studies referenced above raise concerns about the limitations on autonomy and decision latitude afforded to interpreters [5,28,29,33]. Those limitations are often attributed to FCC policy when, in fact, they are policies of the VRS companies [5,48]. It is unclear whether these misattributions (or misinterpretations) are the result of VRS employee ignorance or if this lack of clarity is intentionally reinforced by VRS companies [5]. In any case, it is certainly a convenient confusion, in that the interpretation of unpopular regulations as originating from the FCC and, therefore, being unable to be changed by the VRS companies will directly benefit these companies’ profitability.

One such example is the oft-cited *10-min in-call replacement rule.* This rule asserts that an interpreter must accept and process incoming calls for at least ten minutes before determining whether the call should be transferred to another interpreter [5,40,48]. This rule presumably counters National Association of the Deaf—Registry of Interpreters for the Deaf (NAD-RID) Code of Professional Conduct, specifically, the ethical mandate of tenet 2.0, “Interpreters accept assignments using discretion with regard to skill, communication mode, setting, and consumer needs” [49].

In other words, a VRS interpreter, in deference to their ethical code, might quickly determine poor suitability between their abilities and a caller’s service needs, based on communication mode, setting, or consumer expectations. It is indeed possible to know within the first few seconds that communication would be compromised based solely on how the deaf person is signing, including the use of unfamiliar or regional signs. Or poor suitability could be quickly determined by the topic of the evolving conversation—for example, an interpreter accepting a call from someone (either deaf or hearing) stating they are calling their financial advisor who will be reviewing options for their retirement portfolio. The VRS interpreter may not be adequately knowledgeable about this complicated topic and its vocabulary. In keeping with their ethical code, this should be sufficient reason to transfer the call to a more suitable colleague.

However, many VRS interpreters seem to believe (or have been told) that the FCC requires them to stay on any call for at least ten minutes before they are permitted to transfer the call to another interpreter, even if they recognize that they will be, or *are* being, ineffective [33,40,48]. Given this apparent source of confusion and concern, the FCC issued an order clarifying the rule, stating, “there may be VRS calls during which the party using sign language, the [interpreter], or both, find that they are unable to communicate effectively because of regional dialect differences, lack of knowledge about a particular subject matter (e.g., a technical or complex subject matter), or other reason. In these circumstances, when effective communication is not occurring, we conclude that the 10-min in-call replacement rule is not violated if the VRS provider has another [interpreter] take over the call” [50]. There is evidence that the misinterpretation of the 10 min rule continued for at least a decade [5,29,33] and apparently persists today (see current data).

It is unclear why the 2006 FCC update did not resolve such confusion or why it did not lead companies to update their policies. It might be that VRS companies are insufficiently motivated to recognize or disseminate that clarification. The time a caller is in the queue awaiting an interpreter who is a suitable match is time that the company cannot count as billable. Callers waiting in a queue not only represent lost profits; they may also raise red flags to the FCC. The FCC has set a call–answer benchmark companies must meet—80% of all calls must be answered within 120 s [51].

Even with the provisos offered by the FCC in the above quote, VRS interpreters would be aware that transferring a call is one type of negative efficiency benchmark. Transferring calls is neither efficient nor profitable. This raises the question as to whether VRS interpreters would be likely to use this transfer discretion out of concern that their ranking, and the rewards linked to that ranking with their employer, would suffer.

### 1.5. From Research to Practice

Most research studies of remote and VRS interpreting cited above conclude with proposed changes and improvements, such as the following:

- Improved training and education for interpreters including
  ○ Linguistic and interpreting skills [52];
  ○ Customer service skills [34];
  ○ Working with diverse populations [29];
  ○ Managing the overall expectations and stress of VRS [6,29,37];
  ○ Troubleshooting technology [6,34,37];
  ○ Ethical decision-making [33,52].
- Awareness training and education for VRS users [5,29,34,37].
- Awareness training and education for VRS company management on experiences of microaggressions against marginalized social groups [29,30].
- Routing content-specific calls (e.g., mental health) to specially trained VRS interpreters [28].
- Instituting the use of supervision and case conferencing to give VRS interpreters a confidential place to off-load the effects of challenging calls, as well as share and improve best practices [12,33,53].
- Allowing more opportunities for interpreters to obtain contextual information before placing the call [31,34].
- Ergonomic improvements to physical working conditions and the technology [6,34,37,40].
- Reducing call volume, more time in between calls, and longer breaks [28].
- An incremental, graduated increase in hours for novice VRS interpreters [28].
- Reinstituting the use of privacy screens [28].
- Strategies to reduce the potential for vicarious trauma and musculoskeletal disorders [40].
- Better agreement on the roles and responsibilities of VRS interpreters, especially company policies that counter professional norms [5,33,40].

These examples culminate in the need for broader, systemic changes to VRS policies and practices [12,28,29,31]—a call to action of sorts [31]. Given the deleterious health consequences of cortisol dysregulation, Pollard and colleagues called on all stakeholders to consider a full VRS job redesign based on the occupational health work of Karasek and his demand–control theory. Job redesign involves a review of the demand–control imbalance and requires both an increase in controls (i.e., decision latitude) and a reduction in demands (e.g., call volume) [54]. Pollard and colleagues also called for a consensus planning conference to which all stakeholders would be invited, particularly interpreters themselves who have not had an adequate say in the policies that affected their working conditions [40].

There are several attributes of the prank call viral video mentioned above which serve as relevant context for the current study. While not representative of the vast majority of deaf and hearing VRS callers, abuses do happen. Some are disruptive, some are disturbing and harmful, and some are unequivocally illegal. Furthermore, the prank call video highlights the direct engagement between callers and interpreters before or after a call is connected. Several studies reported on the sometimes-problematic nature of that engagement [5,28,29]. These studies, along with over a decade of anecdotal reports, led our research team to consider the extent, frequency, and consequences of disruptive, abusive, and even illegal calls, including what recourse, if any, VRS interpreters have or use in response to these calls.

## 2. Materials and Methods

The research team developed and trialed several versions of a survey designed to document the types, frequency, and consequences of problematic calls VRS interpreters receive. After obtaining feedback from VRS interpreters who volunteered their time to provide input on these topics, we created and revised an online version of the survey using the Qualtrics survey platform. Our aim in trialing iterative surveys was to ensure that we captured the majority of problematic call types. We did not use this iterative approach to determine the reliability or validity of the survey items.

The survey was approved by the Institutional Review Board (IRB) of the Rochester Institute of Technology (RIT) in New York (USA). It was shared with prospective participants via email and social media platforms. The survey was anonymous with a separate link used to collect email addresses for those opting to participate in a raffle of ten USD 25 Amazon gift cards.

### 2.1. Online Survey

After participants consented to participate in this study, the survey presented a series of demographic questions including the respondent’s tenure of VRS, the average number of hours they typically worked per week, and what days and shifts they typically worked. Then they were presented with a series of yes/no questions regarding the types of calls queried. The types of calls the research team was interested in ranged from rude behaviors (e.g., callers’ making derisive comments to interpreters about their physical traits) to calls where interpreters conveyed content regarding potential illegal activities (e.g., drug deals, prostitution) to exploitative or abusive behaviors (e.g., callers making sexually explicit comments to interpreters). Below is a list of the yes/no questions presented to all respondents:

Have you ever been

- Hung up on by a VRS caller (deaf or hearing) after the call was connected?
- Asked to be transferred to another VRS interpreter?
- Yelled at, scolded, admonished, or sworn at by a caller?
- Involved in a phone call where the callers shared sexually explicit material between themselves that you were expected to interpret?
- Involved in a phone call where a caller made a sexual comment to you or about you?
- Involved in a phone call where you considered that the callers may have been engaging in a drug deal?
- Involved in a phone call where you considered that the callers may have been engaging in arranging prostitution?
- Involved in any calls where you have felt uncomfortable or have provoked negative emotions based on what you were seeing or hearing (including things that were in the background)?
- Witness to poor treatment, neglect, or abuse of a child, elder, or other vulnerable individuals?

If respondents answered yes to any of the above questions, they were presented with two follow-up queries regarding how frequently those types of calls occurred in a week (followed by a Likert scale ranging from rarely to very often) and how processing those types of calls affected them (followed by a free-write text field).

At the end of the survey, respondents had an opportunity to report on any other type of problematic call content not covered in the preceding questions—calls that involved verbal, sexual, or psychologically upsetting content or calls where they were led to feel uncomfortable, taken advantage of, abused, or victimized (free-text field responses). Lastly, they were asked if they reported any of the calls they deemed problematic to their management team, the FCC, or another authority (e.g., police) and what happened as a result of those reports.

### 2.2. Dissemination Barriers

The research team ran into several barriers to disseminating the survey. We were told both directly (e.g., in an email from one company’s management team and in the comment section of social media posts) and indirectly (e.g., our survey links being removed or deleted) that we could not collect this type of data because the questions fell under what the FCC terms “call content.” Call content, or what is relayed between callers is confidential and should not be disclosed by VRS interpreters [55]. As such, all of the follow-up, free-response questions were optional. Many participants opted not to report specific examples of call content. However, the very aim of this study was to uncover some of the abuses and illegal behaviors that are perpetuated because they are shrouded by the FCC’s confidentiality mandate. We consulted with RIT’s IRB to discuss any ethical or legal concerns in light of these challenges and were advised to make some revisions to the consent form to be explicit about the possible risks of survey participation and our efforts to protect respondents’ anonymity. However, we did not alter any of our survey questions in response to this challenge and were not advised to do so by the IRB.

Another barrier to collecting data was the many hundreds of fraudulent survey responses we received. These were so numerous that we closed an initial version of the survey in order to add further security measures to protect against bot and other fraudulent responses. The second version of the survey, with these even greater security measures, still amassed hundreds of fraudulent responses. At our request, the Qualtrics service agents spent considerable time helping us remove likely fraudulent responses. For example, many completed surveys were determined to be fraudulent if the respondent chose the first option to each of the survey questions even when some of the questions required multiple choices.

## 3. Analysis and Results

### 3.1. Participants’ Demographics

After cleaning our original data set, 350 participants remained in our sample. In total, 307 participants responded to all survey questions. The participant sample is highly representative of the Registry of Interpreters for the Deaf (RID) demographic breakdown by ethnicity and is shown in Table 1 below [56]. The RID serves as the governing body for American Sign Language interpreters with nearly 14,000 members. Participants not listed in Table 1 include 17 (5%) who identified as two or more races, 15 (4%) who chose not to report their race, and 4 who did not report their gender.

In total, 315 (90%) respondents held national certification credentials (i.e., National Interpreter Certification, Board for Evaluation of Interpreters, Educational Interpreting Performance Exam), and 35 (10%) did not.

Participants worked between 8 and 40+ h per week, with the most common weekly hours ranging from 8 to 12 (23%) and the least common being over 40 h a week (2%). Because VRS is a 24 h, year-round service, shifts were broken down into 4-h increments beginning at 12:00 am. The most common shift selected by participants was 12:00 p.m. to 4:00 p.m. (32%), and the least common was 12:00 a.m. to 4:00 a.m. (2%). Most interpreters selected Monday as a typical workday (18%), and the least common was Sunday (7%). The sample’s minimum VRS tenure was 2 months, and the maximum was 32 years, with an average of 9.5 years (S.D., 5.89 years).

### 3.2. Survey Results

In total, 340 (97%) of participants reported being hung up on. Of those, 311 (91%) reported they were admonished before the call was terminated.

Participants were asked a series of yes/no questions regarding the call content they had interpreted and had been exposed to including suspected prostitution, drug deals, sexually explicit content, and sexual comments made about the interpreter. If the respondent answered yes to any of these, they were asked how often they were exposed to this call content and if it evoked feelings of discomfort. The response data is outlined below (Table 2).

In total, 153 participants (48%) reported witnessing abuse (defined as poor treatment, neglect, abuse of a child or elder, or other vulnerable individuals) either visually or auditorily while interpreting the call. Furthermore, 147 (47%) respondents claimed there were other psychologically upsetting experiences that were not included in the survey.

The majority of interpreters in this study reported witnessing or experiencing illicit, illegal, psychologically harmful, or otherwise uncomfortable call content. However, when asked about reporting such calls to management or the authorities, 203 (65%) respondents reported a call to management, while only 14 (5%) respondents reported a call to authorities.

### 3.3. Follow-Up Free-Response Questions: Qualitative Data

Not including the demographic sections, there were a total of 20 follow-up, free-response questions. These were presented if the participant responded “yes” to questions regarding certain experiences (e.g., being admonished, exposed to sexually explicit material, witnessing abuse, calls involving illegal activities). Participants were asked to give examples of such call content. Additionally, if they answered “yes” to queries regarding discomfort with processing such calls, they were asked to describe those feelings of discomfort. There were also a series of free-response queries regarding other types of problematic calls that had not already been mentioned.

For the purposes of this article, we will describe a sample of free responses in reference to calls that involved interpreting for sexually explicit and illegal activity. Using content analysis, we collapsed all of the free responses and categorized them into three thematic domains: 1. Access and policy directives; 2. Exposure to and involvement in explicit content; and 3. Types and levels of discomfort. The first category (i.e., access and policy directives) was created based on several spontaneous comments that came in response to other questions regarding the importance of deaf and hard-of-hearing Americans’ rights to access telecom services in the US.

Regarding access and policy directives, several participants noted their support for regulations and policies that govern VRS. For example, participant 208 wrote, “This is my job. Anyone is allowed to make a call that’s sexual.” Similarly, participant 215 dismissively wrote, “Call content is not abuse.” Several other participants highlighted rules about not disclosing call content. One participant stated, “This information is pertaining to call content and therefore I do not feel comfortable answering.” Another participant described following protocol when faced with unwanted comments: “If comments persist, I have offered to transfer the call.” Other comments in this category were those citing concerns VRS interpreters expressed for being effective and doing a good job, even in light of translating explicit content. One participant wrote, “I wasn’t sure how […] I should interpret—it involved moaning and climaxing.”

The second category of participants’ free responses involved exposure to and involvement in explicit content. Many of the examples given were interpreting calls made between intimate partners, from flirtatious remarks to actual phone sex (examples offered by 14 participants). Sometimes these calls were placed to or from jails or prisons (examples offered by eight participants). Other examples included calls to phone sex hotlines or call-in porn sites (examples offered by eight participants). Some more graphic or disturbing included interpreters seeing or hearing children in the same room as the caller, who would be able to access at least some of the sexually explicit content (reported by one participant); seeing pornography playing on a screen in the background (reported by one participant); seeing or hearing individuals masturbating (reported by three participants); and interpreting conversations about sexual violence (reported by one participant). In several instances, interpreters themselves were the subjects of conversation between hearing and deaf callers (i.e., using or objectifying the interpreter), who were likely aware that protocol requires interpreters to solely relay messages and not to participate in the conversation (examples offered by three participants). Some participants reported they were made to sign or say graphic things so they could be observed doing it, sometimes callers would further inquire how the interpreter signed sexual terms, and some respondents expressed concern for being recorded while signing sexual content (reported by seven participants).

Many participants reported receiving explicit comments about their physical appearance and their vocal qualities, (e.g., “you have a sexy voice” or “you sound hot”). They reported being told that they are pretty, big breasted, or asked about their location or dating/marital status (examples offered by 25 participants). Other calls of a sexually explicit nature involved directly soliciting the interpreter, for example, asking the interpreter to engage in “dirty talk” or oral sex, or exposing themselves to interpreters (examples offered by seven participants).

The final free-response category focused on interpreters’ reports on how they felt about interpreting for sexually explicit and illegal activity. Fifty-eight (58) participants opted to answer these free-response questions. Most comments (54) were made about the sexually explicit content in which participants reported feeling uncomfortable (28), disgusted (15), and violated (13). Only a few comments (4) pertained to drug deals in which respondents expressed their discomfort.

### 3.4. Analyses of Sexually Explicit and Illegal Behaviors

While the broad aim of this study was to document the types, frequency, and emotional consequences of problematic calls, we were particularly interested in call content that was sexually explicit or illegal in nature. As previously described, some survey questions dealt with sexually explicit conversations between parties, sexual comments directed at the interpreter, drug deals, prostitution, witnessing potential harm to vulnerable individuals, and whether the interpreter reported such calls to authorities.

Not only do such calls raise concerns for VRS interpreters’ well-being, but they also raise legal concerns if interpreters might be judged as complicit in illegal activity and raise similar legal concerns for their employers and the FCC, which funds the service. As such, the research team further analyzed the survey data to determine not only frequency but the likelihood of receiving and relaying these types of calls.

Our first-level analysis was a simple dichotomous exploration of the frequency with which some of these more compelling VRS calls occurred. Of our participant sample, 251 (79.4%) reported interpreting sexually explicit information between call parties; 216 (69.2%) reported having sexual comments directed to them; 241 (77.7%) reported calls involving drug deals; and 102 (33.0%) reported calls involving prostitution. More than half the sample (52.5%) reported witnessing (visually or auditorily) poor treatment, neglect, or abuse of a vulnerable individual. Despite the high frequency of interpreting for these disturbing and likely illegal calls, only 14 participants (4.7%) reported these experiences to authorities such as federal or state law enforcement.

We next used multivariate logistic regression with the dependent variable being coded “yes” or “no”, including in these analyses the number of months an employee had worked in VRS; the number of VRS hours worked per week; self-reported gender; whether they identified as an underrepresented class; worked the day, night, or overnight VRS shifts; and the day of the week they engaged in VRS work. We did not include national certification in this analysis, as there was little variability in that answer. Table 3 reflects the intersectionality of the participants (gender, race, tenure, etc.) and the odds ratio of them experiencing one of these potentially traumatic calls.

To aid in interpretability, logistic regression results were converted to predicted probabilities using varying scenarios (i.e., continuous predictors were examined at high levels [mean + 1 standard deviation], mean, and low levels [mean − 1 standard deviation]; dichotomous categorical predictors were examined at both levels [no, yes] of the statistically significant (*p* < 0.05) predictors. Non-significant predictors were held at their respective mean values.

For the “sexually explicit” outcome (a), the predicted probabilities ranged from a low of 32% (i.e., low tenure, low hours, not typically working Thursdays, and not typically working Sundays) to a high of 99% (i.e., high tenure, high hours, typically working Thursdays, and typically working Sundays).

For the “sexually directed” outcome (b), predicted probabilities ranged from a low of 57% (i.e., low tenure, and not typically working Sundays) to a high of 87% (i.e., high tenure and typically working Sundays).

For the “drug deal” outcome (c), predicted probabilities ranged from a low of 47% (i.e., low tenure, low hours, and typically working Wednesdays) to a high of 98% (i.e., high tenure, high hours, and not typically working Wednesdays).

For the “prostitution” outcome (d), predicted probabilities ranged from a low of 7% (i.e., low tenure, being female, not typically working Wednesdays, and not typically working Saturdays) to a high of 79% (i.e., high tenure, being male, typically working Wednesdays, and typically working Saturdays).

For the “abuse” outcome (e), predicted probabilities ranged from a low of 43% (i.e., high tenure) to a high of 62% (i.e., low tenure).

For the “reporting” outcome (f), there were no significant predictors.

The number of months working was significant in Table 3 for all call-type variables, as would be expected by a longer tenure of VRS experience. For participant involvement in interpreting sexually explicit conversations and drug deals, the number of hours worked per week was significant as well. Participants who identified as men were more likely to interpret calls they believed involved prostitution. Working on Wednesdays and Saturdays was associated with significantly more calls involving prostitution. Working Wednesdays also was associated with significantly more calls involving drug deals. Sunday workers were more likely to be exposed to sexually explicit content between the call parties, as well as experiencing sexual comments directed at the interpreters themselves. None of the variables were significant regarding participants reporting the experiences queried to authorities.

To further explore the data for intersecting identifiers, we explored the shifts when people worked, the days they worked, their tenure as VRS interpreters, and other factors. This was an effort to better understand how to utilize the data for organizational training purposes.

We asked if respondents had ever experienced callers making a sexual comment to or about them. When examining this question in relation to the days of the week and times of day the respondent worked, evenings proved the more likely time for this to occur, ranging from about 70% on a Sunday to over 80% on a non-Sunday. Even Sunday daytimes, when such calls were “least” likely to occur, the probability still approached 60%.

When examining data regarding this same question in relation to an interpreter’s tenure working in VRS, we distinguished between those with “low-tenure” (36 months or less), “medium-tenure” (48 to 180 months), and “high-tenure” (180 months or more). As expected, a longer tenure was associated with a greater likelihood that a sexual comment had been made to or about the interpreter, exceeding 70% for both the medium- and high-tenure groups. Even the likelihood of the low-tenure group reporting such calls exceeded 60%. This group was likely younger in age, as well as less experienced than the other two groups. One could speculate that the younger, low-tenure group had received such sexual comments *more frequently* than the longer tenured groups, given the close proximity of the probability results (exceeding 60% and 70%, respectively).

Perhaps due to the intersection of statutory frameworks and corporate policy, as noted above, many VRS interpreters failed to report problematic or stressful calls, even calls involving illegality, to employers or outside authorities, despite the notable frequency of such potentially disturbing calls. However, of the small number of participants who did make such reports, more worked evening versus daytime shifts. The complexity of the federal and state statutes, corporate policy, and personal ethics interacting under high-stress work environments is worthy of greater exploration, especially in light of the biometric studies documenting elevated stress for interpreters in earlier research.

## 4. Discussion

The contribution made by the current study is less *that* abusive behaviors occur in VRS settings (other studies have shown this), but how frequently, to what degree, and how they impact interpreters’ well-being. In this case, degree is defined by severity level —from calls that are simply annoying, to disturbing, to illegal. Furthermore, we have identified patterns of when these types of calls tend to be made and how far into one’s VRS tenure interpreters are likely to encounter them.

Lastly, we discovered that there is very little company-sanctioned recourse and company-level follow-through when VRS interpreters are confronted with and even report these types of calls. This was foregrounded earlier when VRS interpreters were compelled to interpret several 911 prank calls made by the masked signer. As noted, if a VRS interpreter receives the types of calls about which we queried and does not choose to process those calls, they are permitted to transfer the call to another interpreter. But since transfers are tracked and lower one’s efficiency and productivity measures, it stands to reason that interpreters may be reluctant to do so out of concern for the impact transfers have on rewards or penalties (e.g., access to preferred work shifts) from their employers. For women in particular, who represent the vast majority of the VRS workforce, losing access to certain work shifts could result in a forced choice between work and the care of a family member (e.g., childcare or eldercare).

Even though it was highly underutilized or seen as less relevant, some VRS companies have put into place measures to assist VRS interpreters to deal with the effects of a problematic call, including access to a counselor through the company’s EAP [12,29]. VRS interpreters also are permitted to file reports of abusive caller behaviors [5]. Yet, in our study, of the participants who answered the question about making reports, 35% (109) opted not to inform their employer, and 96% (298) opted not to inform an outside authority. For those who did, many stated that they either did not know what happened after the report was made or that nothing happened as a result.

Given the frequency of reports of sexual harassment, witnessing of abuse, and involvement in potential criminal behavior cited by the participants, it is surprising there were not more reports made to employers or authorities. In a free-response question about reporting, one participant stated, “I stopped reporting because I don’t feel supported, and it doesn’t do much good anyway.” The sense of futility expressed in this and other free responses to this question are one potential reason for infrequent reporting. Yet another, quite different reason for failure to report abusive calls also was evidenced in the data—the view of some VRS interpreters about protecting the right of telecom access for deaf and hard-of-hearing individuals regardless of the nature of the call.

For example, one participant wrote, “callers are allowed to live free of judgement—just like hearing individuals can.” As noted earlier, another participant assertively wrote, “Call content is not abuse.” Comments such as these led us to create the access directive category when analyzing the qualitative data. These respondents seemed to express a belief that because deaf and hard-of-hearing callers have a legal right to access to the US’s telecommunications network, they also should be free to say or do anything during such calls. Perhaps this is a common VRS workplace narrative. While some VRS interpreters believe that reporting these calls will not make a difference, others believe callers should have the freedom to do as they choose. The result is the same—many of these stressful, abusive, and even illegal calls go unreported.

Choosing to not report abuse, assuming it will amount to nothing or fear that it will result in retribution, is a common experience among women who experience sexual harassment [57]. Eighty-six percent (297) of our participant sample identified as women. Seventy-four percent (220) reported interpreting for sexually explicit content between callers. When asked if callers ever made sexual comments to or about them, 66% (196) answered “yes.”

According to the National Sexual Violence Research Center, eighty-one percent (81%) of women in the US report experiencing some form of sexual harassment in their lifetime [58]. It is reasonable to suggest that the same statistic could be applied to our participant sample. Accordingly, even with the less graphic forms of callers’ sexually explicit material documented in our data, further psychological harm for these women interpreters is a real possibility.

One of the important findings from our data addressed the issue of tenure in VRS work. We found that exposure to disturbing, abusive, or illegal calls could happen at the earliest stage of one’s tenure. VRS companies are a common employment option for those recently graduated from interpreter training programs. As Peterson [40] noted, “That [VRS employment] is rapidly becoming entry-level work in the field of ASL-English interpretation is troubling” (p. 203).

In the US, the Equal Employment Opportunity Commission (EEOC) seeks to ensure that all workplaces are free from many forms of discrimination and prohibited practices, including sexual harassment. According to the EEOC website, “It is unlawful to harass a person (an applicant or employee) because of that person’s sex. Harassment can include ‘sexual harassment’ or unwelcome sexual advances, requests for sexual favors, and other verbal or physical harassment of a sexual nature” [59].

The EEOC has further protections for employees regarding race, age, and gender. This raises legal questions for VRS workplaces if callers are permitted to make derisive comments about an interpreter employee’s age, gender, race, or sexual orientation. Therefore, VRS companies and the FCC should shield VRS interpreters from incidents of sexual harassment and other prohibited behaviors, which were clearly evident in our and others’ data.

The legal concerns raised by our study go beyond those protections offered by the EEOC. As noted earlier, an onscreen interpreter can view the environments of signing callers, most of whom would likely be calling from their homes. Half of the study participants (153) reported witnessing some form of abuse within the field of view of the camera. VRS employers’ policies dictate that most forms of abuse (or imminent danger such as suicide) are not to be reported to outside authorities, with the exception of sexual abuse or exploitation of a child. This exception is based on federal law. However, mandates that workers report various forms of abuse or suicidality actually vary state by state.

For example, if a VRS interpreter was located in New York State, where interpreters are not mandated reporters, and the deaf caller was calling from South Carolina, where interpreters are mandated reporters, it is unclear to which states’ laws this interpreter would be bound. Under South Carolina’s mandated reporter laws, as a human service provider, failure to report witnessed abuse is considered a misdemeanor and punishable by a fine up to USD 500 or 6 months in jail.

This state-by-state variation could arguably be applied to all potentially illegal behaviors of callers and the involved VRS interpreters. This raises issues of complicity, that is, whether an interpreter would be considered an accomplice in drug deals, prostitution, or even in abortions when the location of the caller(s) and the interpreter could dictate different legal requirements.

## 5. From Research to Practice

A summary of how research can translate into practice to benefit both remote interpreters and their employers was outlined earlier, drawing on research from 11 different studies. It is unnecessary to repeat those empirically based action items here. However, our study corroborates one particular suggestion—the need to route particular calls to those who are sufficiently trained, prepared, and willing to take them. In our survey, we asked if certain call types lead to feelings of discomfort. Some of the study participants answered “no” to those questions. If that is true, then it would be a viable option for such calls to be transferred to a team of willing, prepared, and even specialized VRS interpreters.

Another contribution our study makes is demonstrating the use of statistical processes to determine patterns. These patterns (e.g., the most likely times and days of the week these calls occur) could help VRS companies ensure that trained and willing staff are available. While our data were dependent on the reports made by the survey participants, VRS companies and their ability to better gather, document, and monitor these data could determine such patterns with even greater reliability.

Routing or transferring calls to specialized teams was similar to a suggestion made by Bower [28]. That same year, the FCC accepted a VRS joint-company proposal which sought to increase the use of interpreter teams for difficult calls. The FCC also accepted the joint proposal to employ technologies that route specialty calls (e.g., 911 calls) to specially trained VRS interpreters instead of the current random assignment protocol [60]. A full ten years later, we have no evidence to indicate that those federal allowances, designed to support VRS interpreters, have been instituted. Perhaps Casar’s question, “Is [the money] going to go to the interpreters and their clients…or is it going to go to the profits of a few extra-wealthy people?” is further evidenced by this lack of action.

VRS service users are most prominently deaf and hard-of-hearing sign language users who must have equal rights to the same telecommunication services any American enjoys. Indeed, research into the health and welfare of VRS interpreters by studies such as ours exemplifies this same concern for rights to equal access. All rights come with limits. Just as the right to free speech does not permit someone to falsely yell “Fire!” in a crowded theatre, VRS interpreters must also have the same workplace protections that the EEOC grants. The technology is available today to put some of those protections in place.

## 6. Study Limitations and Future Research

This was a “proof of concept” study. While our previous research has consistently documented high levels of occupational health risk among VRS interpreters, via both well-respected self-report inventories and evidence of cortisol dysregulation, we had not yet studied in detail *why* this employee group was at particular risk. Based on previous research and our team’s direct experience with the VRS occupation, we hypothesized that stress-inducing caller behavior, along with potentially exacerbating employer (and possibly federal) policies, would be fertile topics to explore. As our first foray into examining such potential underlying factors, our choice of the survey methodology we employed has left this matter only partially elucidated. Our study certainly had limitations in that regard.

First, as noted, we encountered significant difficulties simply disseminating our survey and collecting a robust data set. One large VRS company cautioned us not to study the topic of caller behavior and may well have discouraged its employees from participating. Survey links were removed from some online locations we posted to. Data submitted via bots and other obviously fraudulent survey responses required significant effort to address before the final, reliable data set was compiled. Finally, a considerable number of respondents provided only limited data, perhaps stemming from imagined consequences of responding in a more candid manner or other factors.

Respondents’ data were self-reported in nature. In that regard, the generalizability of the data cannot be assured. Cross-validation using VRS companies’ extensive data collection mechanisms, noted above, would greatly enhance a future study of this type. Whether VRS companies would be willing to collaborate in such an endeavor is questionable, as we learned via the impediments we encountered to our survey dissemination efforts and via the comments of some of our survey respondents. The qualitative data was necessarily more subjective. Our findings in this regard would be strengthened in future studies via employing larger subject samples, focus groups, psychological testing, or other methods—especially if interpreter participation was encouraged and sanctioned by VRS employers who stand to benefit from a healthier workforce.

Of note, our focus on stress, even trauma, in VRS work may well have inhibited some respondents’ willingness to address such issues. Prior trauma exposure or individual coping capacity may have influenced disclosure to the survey questions. Some studies exploring issues of trauma include resources such as contact information to relevant mental health services. We did not do so in this study, given the option for respondents to simply not respond to discomforting questions. A future study employing supportive safeguards could yield more, even distinct, disclosure dynamics.

Additional suggestions for future research on the topic of VRS interpreter health risks, and the potential remediation thereof, were called for in this publication. They included convening a consensus planning conference bringing together a truly representative array of stakeholders in the VRS enterprise. Also, rigorous efforts at VRS job redesign such as Karasek’s model [55] could be implemented and studied. A smaller-scale example of a job redesign research study would be to implement VRS call-routing policies to specially trained interpreters with medical, trauma, law enforcement, or other competencies which could potentially and significantly reduce the stress load on otherwise generally trained interpreters. As noted, the FCC has endorsed such an idea in a joint company proposal [60], but, to our knowledge, this has not been implemented nor have its results been studied. Given the global presence of the two main VRS companies, an international study could investigate both the experiences of VRS interpreters and the uniformity of company policies worldwide.

## 7. Conclusions

While a high degree of occupational health risk among VRS interpreters has been repeatedly demonstrated in previous research, the present study advances this scholarship by shedding light on certain factors underlying that risk. A notable degree of abusive and otherwise stressful caller behavior was documented by our survey respondents. Calls involving sexual content, drug deals, and prostitution were reported, as were VRS interpreters themselves being the subject of sexual and derisive comments and even bearing witness to callers’ abusive behavior toward vulnerable individuals. Our data documented patterns regarding when such calls were more likely to occur, how they affected our respondents, and the rather infrequent incidence of such calls being reported to employers or outside authorities. A sense of futility in making such reports was noted by some respondents, while others expressed belief that call content should not be constrained, regardless of its nature.

Beyond problematic call content per se, our data substantiated the finding that VRS company policies and practices exacerbate stress-related occupational health risks of their employees. These include economic pressures leading to interpreter reluctance to transfer distressing calls, since doing so can negatively impact their productivity metrics and access to preferred work shifts, a likely factor for the predominantly female VRS workforce. Lack of clarity regarding the distinction between VRS company policies and FCC policies is another barrier to the potential remediation efforts called for in this publication. There is a compelling need for a consensus planning conference involving a broad array of stakeholders in the VRS enterprise. The FCC and VRS companies should be represented but also deaf and hearing consumers of the service, and especially VRS interpreters themselves. This large, at-risk workforce rarely has effective opportunities to attest to experiences contributing to occupational health risks and participate in crafting effective solutions through job redesign.

Technology has and will always be an important vehicle for the provision of interpreting services. It is important to interpreters’ well-being when working in conjunction with technology that employers address patterns of concerns that emerge empirically. It has been well over a decade since research studies into VRS interpreting began highlighting multiple areas of concern with very little response or change at the federal or corporate level. In the absence of sorely needed reforms, VRS companies and their employees face profound legal questions as well. These include potential complicity in illegal activities, unclear responsibilities for compliance with state-mandated reporter laws, and compliance with EEOC protections against sexual harassment and discrimination in the workplace.

## Figures and Tables

**Table 1:** Study sample vs. RID membership demographics.

Study Sample vs. RID Membership Demographics							
	White	African American	Hispanic/Latinx	Pacific Islander/Asian American	Men	Women	Nonbinary
Sample	86% (302)	3% (12)	6% (20)	0.5% (2)	12% (42)	85% (297)	2% (6)
RID	83%	6%	7%	2%	14%	86%	0.15%

**Table 2:** Interpreted illicit content.

Illicit Content				
Response	Interpreted Drug Deal Calls	Interpreted Prostitution Calls	Interpreted Sexually Explicit Material	Sexual Comments About the Interpreter
No	73	215	70	100
Yes	253	110	262	228
Very Often	6	0	4	4
Often	20	4	11	18
Sometimes	87	15	76	65
Rarely	139	89	167	140
Discomfort with Call	71	110	162	195

**Table 3:** Logistic regression results involving… (**a**). Prediction of ever being involved in a phone call where the callers shared sexually explicit material between themselves that the interpreter was expected to interpret (n = 316). (**b**). Prediction of ever being involved in a phone call where a caller made a sexual comment to the interpreter or about the interpreter (n = 312). (**c**). Prediction of ever being involved in a phone call where the interpreter considered that the callers may have been engaging in a drug deal (n = 310). (**d**). Prediction of ever being involved in a phone call where the interpreter considered that the callers may have been engaging in prostitution (n = 309). (**e**). Prediction of ever being a witness to the poor treatment, neglect, or abuse of a child, elder, or other vulnerable individual (n = 301). (**f**). Prediction of ever reporting any of these calls to another authority (e.g., police, the FCC, etc., authority other than manager at company) (n = 296).

(**a**) **Interpreting Sexually Explicit Material (n = 316)**				
**Variable**	**b**	***p***	**OR**	**95% OR CI**
Intercept	−1.620	0.068		
Months of VRS work	0.014	<0.001	1.015	1.009–1.020
Hours/week in VRS	0.257	0.078	1.292	1.110–1.505
Male	0.581	0.550	1.787	0.608–5.249
Minority	0.451	0.497	1.570	0.593–4.159
Overnight shift	1.276	0.689	3.582	0.929–13.813
Daytime shift	−0.766	0.686	0.465	0.121–1.782
Evening shift	0.414	0.346	1.512	0.768–2.978
Mondays	0.427	0.478	1.532	0.600–3.914
Tuesdays	−0.573	0.452	0.564	0.233–1.367
Wednesdays	−0.759	0.439	0.468	0.198–1.107
Thursdays	−0.937	0.435	2.551	1.088–5.985
Fridays	0.441	0.416	1.550	0.688–3.512
Saturdays	0.620	0.403	1.858	0.843–4.097
Sundays	0.913	0.458	2.491	1.015–6.109
(**b**) **Sexual Comments to/About the Interpreter (n = 312)**				
**Variable**	**b**	***p***	**OR**	**95% OR CI**
Intercept	0.625	0.337		
Months of VRS work	0.004	0.020	1.004	1.001–1.008
Hours/week in VRS	0.084	0.156	1.088	0.968–1.222
Male	−0.516	0.168	0.597	0.287–1.242
Minority	0.282	0.466	1.326	0.621–2.832
Overnight shift	0.229	0.576	1.257	0.563–2.806
Daytime shift	0.257	0.584	1.293	0.516–3.244
Evening shift	0.492	0.284	1.636	0.937–2.856
Mondays	0.175	0.395	1.191	0.549–2.583
Tuesdays	0.144	0.377	1.155	0.551–2.419
Wednesdays	−0.491	0.175	0.612	0.301–1.245
Thursdays	−0.298	0.417	0.742	0.361–1.524
Fridays	0.274	0.421	1.315	0.675–2.562
Saturdays	−0.137	0.317	0.872	0.469–1.622
Sundays	0.968	0.008	2.633	1.293–5.361
(**c**) **Interpreted Possible Drug Deals (n = 310)**				
**Variable**	**b**	***p***	**OR**	**95% OR CI**
Intercept	−0.434	0.567		
Months of VRS work	0.016	<0.001	1.016	1.011–1.022
Hours/week in VRS	0.178	0.014	1.194	1.036–1.377
Male	−0.608	0.465	0.544	0.219–1.354
Minority	−0.805	0.418	0.447	0.197–1.014
Overnight shift	1.050	0.066	2.856	0.935–8.727
Daytime shift	−0.150	0.783	0.861	0.295–2.507
Evening shift	−0.194	0.591	0.824	0.406–1.671
Mondays	0.606	0.169	1.832	0.773–4.342
Tuesdays	0.358	0.423	1.431	0.596–3.438
Wednesdays	−1.003	0.030	0.367	0.148–0.909
Thursdays	−0.318	0.482	0.727	0.299–1.767
Fridays	−0.058	0.893	0.944	0.405–2.200
Saturdays	0.442	0.272	1.556	0.707–3.425
Sundays	−0.123	0.415	0.885	0.392–1.997
(**d**) **Interpreted Possible Prostitution Calls (n = 309)**				
**Variable**	**b**	***p***	**OR**	**95% OR CI**
Intercept	−3.251	<0.001		
Months of VRS work	0.010	<0.001	1.010	1.006–1.014
Hours/week in VRS	0.114	0.061	1.121	0.995–1.262
Male	0.899	0.022	2.457	1.140–5.293
Minority	0.597	0.110	0.447	0.874–3.772
Overnight shift	−0.227	0.568	0.797	0.365–1.737
Daytime shift	−0.692	0.144	0.501	0.198–1.267
Evening shift	0.037	0.904	1.038	0.566–1.902
Mondays	0.395	0.357	1.484	0.641–3.435
Tuesdays	−0.718	0.077	0.488	0.220–1.082
Wednesdays	0.794	0.045	2.211	1.018–4.804
Thursdays	0.339	0.388	1.403	0.650–3.028
Fridays	0.541	0.157	1.718	0.811–3.638
Saturdays	0.800	0.013	2.226	1.183–4.186
Sundays	−0.566	0.099	0.568	0.290–1.113
(**e**) **Witnessed Harm to Vulnerable Individuals (n = 301)**				
**Variable**	**b**	***p***	**OR**	**95% OR CI**
Intercept	0.897	0.140		
Months of VRS work	−0.006	0.002	0.994	0.991–998
Hours/week in VRS	−0.099	0.073	0.906	0.813–1.009
Male	0.296	0.423	1.345	0.651–2.776
Minority	0.660	0.069	1.934	0.951–3.933
Overnight shift	−0.119	0.749	0.888	0.429–1.838
Daytime shift	0.133	0.760	1.142	0.488–2.675
Evening shift	0.110	0.693	1.116	0.647–1.923
Mondays	−0.523	0.166	0.593	0.283–1.241
Tuesdays	0.666	0.059	1.947	0.975–3.889
Wednesdays	−0.219	0.511	0.803	0.417–1.545
Thursdays	0.111	0.746	1.117	0.573–2.179
Fridays	0.021	0.948	1.021	0.544–1.916
Saturdays	−0.085	0.774	0.919	0.516–1.637
Sundays	−0.134	0.667	0.875	0.476–1.609
(**f**) **Reported to the Authorities (n = 296)**				
**Variable**	**b**	***p***	**OR**	**95% OR CI**
Intercept	−2.224	0.071		
Months of VRS work	0.001	0.818	1.001	0.993–1.009
Hours/week in VRS	0.071	0.600	1.073	0.824–1.393
Male	−0.180	0.831	0.836	0.161–4.350
Minority	0.228	0.753	1.256	0.305–5.168
Overnight shift	0.621	0.424	1.862	0.405–8.546
Daytime shift	−1.390	0.075	0.249	0.054–1.150
Evening shift	0.551	0.462	1.736	0.400–7.533
Mondays	0.232	0.787	1.261	0.234–6.801
Tuesdays	−0.498	0.495	0.608	0.145–2.540
Wednesdays	0.338	0.756	1.403	0.319–6.177
Thursdays	−0.584	0.411	0.558	0.139–2.245
Fridays	−0.119	0.871	0.888	0.211–3.731
Saturdays	0.345	0.591	1.412	0.401–4.974
Sundays	−1.138	0.135	0.321	0.072–1.424

Note: b = parameter estimate; *p* = *p*-value; OR = odds ratio; 95% OR CI = 95% odds ratio confidence interval.

## Data Availability

The raw data supporting the conclusions of this article will be made available by the authors on request.
